# A Comprehensive Review of Transaminitis and Irritable Bowel Syndrome

**DOI:** 10.7759/cureus.16583

**Published:** 2021-07-23

**Authors:** Eyad Gadour, Zeinab Hassan, Rajaey Gadour

**Affiliations:** 1 Gastroenterology and Hepatology, University Hospitals of Morecambe Bay NHS Foundation Trust, Lancaster, GBR; 2 Department of Medicine, Stockport Hospital NHS Foundation Trust, Manchester, GBR; 3 Responsible Medical Services, The National Ambulance, Abu Dhabi, ARE

**Keywords:** irritable bowel disease, transaminitis, diarrhoea, small bowel bacterial overgrowth, liver transplant

## Abstract

We observed in the literature that irritable bowel syndrome (IBS) may be linked to irregular parameters of the metabolic system (MS) and liver function. For that reason, we conducted this systematic review to comprehensively analyze the association of transaminitis (elevated alanine transaminase (ALT)) with IBS. This review was designed by following methods described in the Cochrane Handbook for Systematic Reviews of Interventions. Published peer-reviewed journal articles were included. Data were extracted based on study design, age, gender, author, date of publication or availability online, publication type, participants, gender (M/F), and types of IBS.

Our electronic multiple databases yielded a total of 519 preliminary studies; we then removed duplicate studies and left with 326 studies. After reviewing the full text of these articles, a total of 83 studies were eliminated and lastly, three studies were selected for this systematic review for quantitative and qualitative analysis. All the enrolled subjects in included studies were diagnosed with IBS by the Rome II and III criteria and among these sub­jects, 50.4% had IBS-D, 13.8% had IBS-C, 30.3% had IBS-M, and 3.5% had IBS-U. The prevalence of elevated ALT with other liver enzymes (γ-GT levels and aspartate aminotransferase (AST)) in patients with irritable bowel syndrome whether their body mass index (BMI) was high or not (16.9% vs. 7.7%; *p*=0.015) and γ-GT (24.1% vs. 11.5%; *p*=0.037), Lee et al., 2016. The IBS-D subtype was seen more commonly in patients whose alcohol intake was significantly high however their study data showed no significant change in elevation of ALT. The upper limits normal values for serum liver enzymes were de­fined as 41 international per liter in males and 31 international units per liter in females for ALT. No significant relationships were observed between IBS status and elevated γ-GT (OR, 1.647; 95% CI, 0.784-3.461).

The review study proposes a potential relation between elevated ALT levels, MS, and IBS, and this review might be the first review in IBS patients to observe the association of elevated ALT in the IBS population. Although further additional trials with a large sample size will be required to confirm these results. Furthermore, for assessing the efficacy of the manipulation of gut microbiota ran­domized controlled trials in a large population of IBS patients are needed to establish a causal-resultant relationship between IBS, MS, and liver damage.

## Introduction and background

Irritable bowel syndrome (IBS) is a chronic gastrointestinal tract disorder that is described as abdominal discomfort, alteration of bowel routine, and abdominal pain [1]. Strong relationships between gut microbiota and metabolic pathways have been revealed in recent studies [2]. IBS is the most diagnosed and observed disorder in the general population; the prevalence of this disease in the worldwide population is approximately 8%-35%, and in the UK, the prevalence of IBS is 17% in which the female population covers 23% and men 11% [3-4]. Recent developments in medicine made it possible to observe the pathophysiology behind the development and manifestation of IBS, which includes dysbiosis, previous gastrointestinal (GI) infection, altered levels of GI hor­mones, vis­ceral hypersensitivity, dysregulation of the brain-gut axis, alteration in the autonomic nervous system, and abnormal overgrowth of gut microbiota in the GI tract [5]. With the features of disordered defecation, IBS is associated with abdominal discomfort and pain, which has been recently defined by the Rome III criteria as a functional disorder of the GI tract [6].

Several hypotheses proposed that gut permeability could be the reason for altered gut micro­biota in the small intestine [7]. In their experimental study in animal models, Brenner and Schnabel demonstrated that the onset and development of nonalcoholic fatty liver disease could be caused by the contribution of intestinal microbiota’s translocation of microbial products and progression via a breakdown of the barrier in intestinal lining [8]. Therefore, the pathogenesis of nonalco­holic fatty liver disease (NAFLD) is hypothesized as a probable reason for increased gut permeability [9].

In the general population, alanine aminotransferase (ALT) is used as one of the most routinely measured in the standard process of screening in diagnosing liver diseases like NAFLD [10]. We observed in the literature that IBS may be linked with irregular parameters of the metabolic system and liver function. For that reason, we are conducting this review to comprehensively analyze the association of transaminitis (elevated ALT) with IBS.

## Review

Materials and methods

This systematic review will be designed by following methods described in the Cochrane Handbook for Systematic Reviews of Interventions (Higgins et al., 2019) ("Higgins JPT, Thomas J, Chandler J, Cumpston M, Li T, Page MJ, Welch VA (editors) [11], Cochrane Handbook for Systematic Reviews of Interventions. 2nd Edition. Chichester (UK): John Wiley & Sons, 2019.," 2019) as well as guidelines presented in the Preferred Reporting Items for Systematic Reviews and Meta-Analyses (PRISMA) checklist (Moher et al., 2009) [12]. A PICOT (patient, intervention, comparison, outcome, and time) question was generated to guide the review and clinical question.

Search Strategy

Electronic searches: Detailed search strategies for each electronic database will be developed. These will be based on the one used for Pubmed, Medline, CINAHL, Cochrane, and Google Scholar but with appropriate database-related search strategies modification such as the use of truncations, wildcards, and filters.

Searching other resources: The references of all the included studies were checked and we used the citation alert to search for more up-to-date publications or new studies.

**Figure 1:**
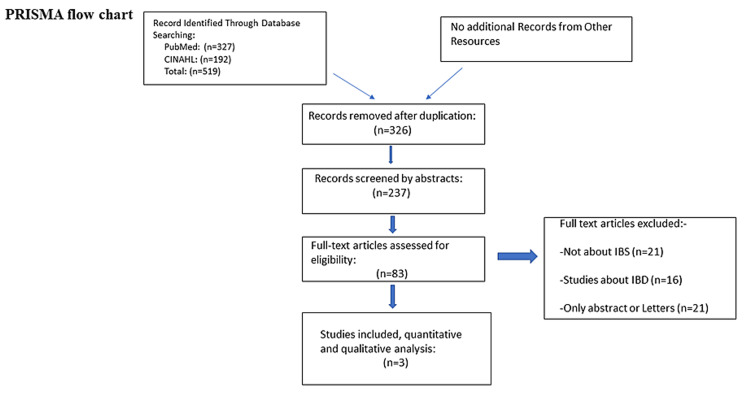
PRISMA flow chart PRISMA: Preferred Reporting Items for Systematic Reviews and Meta-Analyses

A flowchart of the literature search used is shown in Figure 1.

Search terms: Search terms included “Irritable bowel syndrome”,” IBS, “SIBO”, “alanine aminotransferase’’, “transaminitis”, ‘‘ALT’’, and ‘‘metabolic syndrome’’.

We used MeSH terms, including irritable bowel syndrome, IBS, and alanine aminotransferase, transaminitis, ALT - a MeSH heading in PubMed.

Subject headings in CINAHL included irritable bowel syndrome, transaminitis, IBS and alanine aminotransferase, and transaminitis.

Inclusion and Exclusion Criteria

Published peer-reviewed journal articles were included if they met the following criteria: (1) If the study was original research of qualitative and quantitative studies examining the relation of elevated liver enzyme ALT with IBS; (2) Studies published in English; (3) Had empirical data on the four types of IBS with the four classifications -constipation, diarrhea, mixed, and unclassified.

Study Selection

Two of the reviewers screened the titles and abstracts of articles retrieved through the electronic search and found full-text articles for relevant studies. After the exclusion of irrelevant studies, the remaining studies were assessed for relevance as full texts. The same reviewers also manually scanned the reference lists of relevant studies for the secondary search. All reviewers assessed all relevant studies against the inclusion criteria.

Data Extraction

General information: Data were extracted based on study design, age, gender, author, date of publication or availability online, publication type, participants, gender (M/F), and types of IBS. The details of study characteristics are presented in Table 1.

**Table 1:** Baseline demographic characteristics of included studies ALP: Alkaline Phosphates; ALT: Alanine Aminotransferase; AST: Aspartate Aminotransferase; BMI: Body Mass Index; F: Female; GGT: Gamma-Glutamyl Transferase; M: Male; SD: Standard Deviation; VS: Versus; WBC: White Blood Cell

Study ID	Country	Enrolled Study Population (case/total)	Gender (Female/Male)	Age (range, mean ± SD)	Comparison	Follow-up (years)	Adjusted covariates
Lee et al., 2016 [13]	Korea	343	260/83	42.3±5.3	Highest quintile vs lowest quintile (29 vs. 14)	7 (6.5-6.9)	Age, alcohol intake, cigarette smoking, family history, regular physical activity, WBC count, GGT, ALP and AST.
Fouad M et al., 2010 [14]	Egypt	259	181/77	44.7±7.1	Highest quintile vs lowest quintile (M:19.5 vs. 33.4; F:13.5 vs. 21.3)	3	Age, alcohol intake, physical activity, smoking habits and GGT.
Khayyatzadeh et al., 2017 [17]	Iran	865	772/193	14.4±1.4	Highest quintile vs lowest quintile of ALT per SD increment of log ALT level	5.2 (4.5-6.6)	Age, sex, ethnicity, clinical center, alcohol intake, waist circumference, and BMI.

Study Characteristics

Setting; study design; study inclusion and exclusion criteria

Results

Our electronic multiple databases yielded a total of 519 preliminary studies, we then removed duplicate studies and left with 326 studies. By initial screening of the titles and abstracts, a further 237 articles were removed and 89 articles were filtered out for full-text screening. After reviewing the full text of these articles, a total of 83 studies were eliminated and, lastly, three studies (Lee et al., 2016, Fouad et al., 2010 and Khayyatzadeh et al., 2017 [13-15]) were selected for this systematic review for quantitative and qualitative analysis. Figure 1 shows the utilization of the PRISMA 2009 flow diagram, demonstrating the process of screening these articles. While going through this phase of our systematic review, we carefully followed the guidelines of the PRISMA statement [16].

Study Characteristics

All three of our included studies were performed on human participants of age 14 and older. The study sample sizes ranged from 258 participants to 865 patients. A total number of 1466 patients were admitted in these three included studies for the evaluation of transaminitis in patients with IBS. The main characteristics of all these included studies with descriptions of their included patients are given in Table 1.

All three studies were based on hepatic disorder and its link with irritable bowel syndrome, and both male and female patients were studied in these studies.

Design and Quality Appraisal

The three studies included in this review were retrospective and prospective studies. The studies were analyzed for evidence level and quality utilizing the Johns Hopkins Nursing Evidence-Based guide. The Johns Hopkins guide assigns a level of evidence from I to V based on the type of study. According to the Johns Hopkins guide, studies are further evaluated for quality. Quantitative studies are ranked from A as the highest quality of study to a C, which includes a study that is of low quality or contains major flaws. A few of the important components of an A-level quality study include “constant, definitive conclusions; sufficient sample size for the study design; generalizable results; adequate control; and consistent recommendations based on a comprehensive literature review that covers systematic indication to scientific evidence. The three studies included in this review were all retrospective and prospective trials and therefore are assigned to a level I of evidence. All studies utilized double blinding to minimize any bias in their results and therefore were assigned an A for quality. To minimize this bias risk, the outcome assessors were blinded. Quality assessment of included studies is presented in Table 2 below.

**Table 2:** Quality assessment of included studies

Study ID		1	2	3	4	5	6	7	8		9	10	11	12	13		14	Study Quality
Lee et al., 2016 [13]		Yes	Yes	Yes	No	No	Yes	Yes	No		Yes	No	Yes	No	NA		No	Good
Fouad M et al., 2010 [14]		Yes	Yes	Yes	No	No	Yes	Yes	No		Yes	No	Yes	No	NA		Yes	Good
Khayyatzadeh et al., 2017 [17]		Yes	Yes	Yes	No	No	Yes	No	No		Yes	No	Yes	No	NA		Yes	Good

Findings

All of the enrolled subjects in included studies were diagnosed with IBS by Rome II and III criteria and among these sub­jects, 50.4% had IBS-D, 13.8% had IBS-C, 30.3% had IBS-M, and 3.5% had IBS-U demonstrated in Table 3. The mean length of follow-up ranged from three to seven years. Mostly one test was used by all studies to diagnose the bacterial overgrowth of the intestinal tract, which is the main cause for the manifestation of IBS. The study by Lee et al. used LHBT with intestinal aspirate culture to identify the cause [13]. Another study by Khayyatzadeh et al. used aspirate culture LHBT and GHBT [15]. One other study used the culture test for duodenal aspirate with GHBT [17]. Not all the studies took patients with the same type of IBS, one study took patients with diarrhea type predominantly and the other study preferred constipation type for their evaluation [14,17].

**Table 3:** IBS and its types, with the prevalence percentage IBS: irritable bowel syndrome

Study ID	IBS-C	IBS-D	IBS-M	IBS-U
Lee et al., 2016 [13]	4/16 (25.0%)	10/24 (41.7%)	4/25 (16.0%)	11/51 (21.6%)
Fouad M et al., 2010 [14]	16/51 (37.%)	15/38 (39.5%)	6/48 (12.5%)	3/21 (14.3%)
Khayyatzadeh et al., 2017 [17]	6/19 (31.6%)	21/35 (60.0%)	15/58 (25.9%)	10/27 (37.0%)

Lee et al. reported the prevalence of different subtypes of IBS in patients with using GHBT [13]. SIBO was more common than the other subtypes, especially with diarrhea-predominant IBS. They also reported the prevalence of elevated alanine aminotransferase (ALT) with other liver enzymes (γ-GT levels and AST) in patients with irritable bowel syndrome whether their BMI was high or not. the IBS-D subtype was seen more commonly in patients whose alcohol intake was significantly high however their study data showed no significant change in elevation of ALT. In their study, the upper limits normal values for serum liver enzymes were de­fined as 41 international per liter in males and 31 international units per liter in females for ALT. However, compared to subjects without IBS, those with IBS had significantly higher values for BMI, waist circumference (WC), ALT, alkaline phosphate (ALP), γ-GT, total cholesterol (TC), triglyceride (TG), and low-density lipoprotein (LDL) cholesterol.

Discussion

Our current systematic review showed a significantly higher prevalence of elevated ALT levels and AST in IBS patients compared to the normal non-IBS group in the studies. After controlling for potential confounding factors, the relationship remained statistically significant. The higher prevalence of small intestinal bacterial overgrowth (SIBO) and altered gut microbiota in IBS patients has been reported in previous studies which probably creates a relation between altered gut SIBO, microbi­ota, and IBS [18-20]. Several hypotheses proposed that gut permeability could be the reason for altered gut micro­biota in the small intestine [7,21]. Brenner and Schnabel demonstrated in their experimental study on animal models that the onset and development of nonalcoholic fatty liver disease could be caused by the contribution of the intestinal microbiota’s translocation of microbial products and progression via a breakdown of the barrier in intestinal lining [8].

Therefore, the pathogenesis of NAFLD is hypothesized as a possible reason for increased gut permeability [9]. In the general population, ALT is used as one of the most routinely measured in the standard process of screening in diagnosing liver diseases like NAFLD.

Increased lev­els of endotoxin and tumor necrosis factor-alpha and increased intestinal permeability could support the evidence of a pathogenic role for altered gut mi­crobiota in IBS patients with hepatitis or NAFLD. Liver damage may occur due to an increase in nitric oxide-related substances and a boost in the production of pro-inflammatory cytokines, which are induced by gut-derived endotoxins that activate the Kupffer cells; this damage to the liver results in the release of ALT that is primarily clustered in the cytosol of the hepatocyte and causes a considerable increase in serum ALT levels [22].

Hence, endotoxins and possibly other gut-derived, pro-inflammatory bacterial products are involved in the development of liver disease, which may help in explain­ing why IBS is associated with elevated ALT levels. Although it has not been investigated in any previous study, further study is needed to confirm this hypothesis of the relationship between liver enzymes and IBS in humans.

The relationship between IBS status and IBS in an adult popula­tion has been assessed by a small number of epidemiological studies up till now, and its fundamental causes of pathophysiologic alterations are still not entirely studied; nevertheless, some specific strains of Lactobacillus or Bifidobacterium in humans, and their anticipated diverse mechanisms in lipid-lowering effects have been studied in several in vitro studies [23]. Patients transplanted for ALT have slightly better overall survival compared to viral etiologies and even though alcohol relapse may be considered a failure in the selection and/or management of the patient, transplantation for ALT is a very effective, utilitarian use of a scarce resource and indeed may be a better use than if used for patients with end-stage HCV. However, we do not have enough information to estimate whether those transplanted for ALT have a greater benefit from transplantation because we do not have a robust measure of survival without transplantation [23]. Bacterial lipopolysaccharides (LPS) derived from gram-negative bacteria residing in the intestinal tract may act as a triggering factor, linking inflammation to high-fat diet-induced MS. The results of human studies have support­ed these findings. Treatment of humans with polymyxin B, an antibiotic that specifically targets gram-negative organisms, was found to reduce LPS expression and hepatic steatosis [24].

It is observed that IBS has an impending adverse effect on serum liver enzymes ALT and its components possibly due to its effect on nutrient absorption, food digestion, or dietary pattern. Nevertheless, a dietary pattern in relationships with IBS was insignificant. According to a previous study, this suggested that IBS is not related to dietary habits and/or nutritional intake [15]. Low-grade mucosal inflammation increased intestinal mucosal permeability, and abnormal intestinal motility is an accepted mechanism that alters the gut function and generates symptoms of IBS regardless of the primary causes of pathophysiologic changes still not being completely understood.

## Conclusions

The review study proposes a potential relation between elevated ALT levels, MS, and IBS, and this review might be the first review in IBS patients to observe the association of elevated ALT in the IBS population. Although further additional trials with a large sample size will be required to confirm these results. Furthermore, for assessing the efficacy of the manipulation of gut microbiota ran­domized controlled trials in a large population of IBS patients are needed to establish a causal-resultant relationship between IBS, MS, and liver damage.
